# Stability and Dynamics of Polycomb Target Sites in *Drosophila* Development

**DOI:** 10.1371/journal.pgen.1000178

**Published:** 2008-09-05

**Authors:** Camilla Kwong, Boris Adryan, Ian Bell, Lisa Meadows, Steven Russell, J. Robert Manak, Robert White

**Affiliations:** 1 Department of Physiology, Development and Neuroscience, University of Cambridge, Cambridge, United Kingdom; 2 Theoretical and Computational Biology Group, MRC Laboratory of Molecular Biology, Cambridge, United Kingdom; 3 Affymetrix Inc., Affy Labs–Transcriptome, Santa Clara, California, United States of America; 4 Department of Genetics, University of Cambridge, Cambridge, United Kingdom; Netherlands Cancer Institute, Netherlands

## Abstract

Polycomb-group (PcG) and Trithorax-group proteins together form a maintenance machinery that is responsible for stable heritable states of gene activity. While the best-studied target genes are the Hox genes of the Antennapedia and Bithorax complexes, a large number of key developmental genes are also Polycomb (Pc) targets, indicating a widespread role for this maintenance machinery in cell fate determination. We have studied the linkage between the binding of PcG proteins and the developmental regulation of gene expression using whole-genome mapping to identify sites bound by the PcG proteins, Pc and Pleiohomeotic (Pho), in the *Drosophila* embryo and in a more restricted tissue, the imaginal discs of the third thoracic segment. Our data provide support for the idea that Pho is a general component of the maintenance machinery, since the majority of Pc targets are also associated with Pho binding. We find, in general, considerable developmental stability of Pc and Pho binding at target genes and observe that Pc/Pho binding can be associated with both expressed and inactive genes. In particular, at the Hox complexes, both active and inactive genes have significant Pc and Pho binding. However, in comparison to inactive genes, the active Hox genes show reduced and altered binding profiles. During development, Pc target genes are not simply constantly associated with Pc/Pho binding, and we identify sets of genes with clear differential binding between embryo and imaginal disc. Using existing datasets, we show that for specific fate-determining genes of the haemocyte lineage, the active state is characterised by lack of Pc binding. Overall, our analysis suggests a dynamic relationship between Pc/Pho binding and gene transcription. Pc/Pho binding does not preclude transcription, but levels of Pc/Pho binding change during development, and loss of Pc/Pho binding can be associated with both stable gene activity and inactivity.

## Author Summary

Cells make fate decisions as they progressively differentiate into specific cell types during development. The stability of these decisions is important and is achieved, in part, by changes to the chromatin that packages DNA in the nucleus. A key set of protein complexes that together constitute the Polycomb-group/Trithorax-group (PcG/TrxG) machinery is involved in chromatin modification and is known to operate at a large number of genes involved in developmental decisions. The PcG proteins establish stable gene repression, whereas the TrxG counteract the PcG to enable gene activation. How this PcG/TrxG balance works is not understood. By mapping PcG protein binding to chromatin in vivo, we show, in general, a relatively constant association of PcG protein at target genes during development. However, we also find changes in binding at specific genes. While some of these changes are consistent with a loss of PcG proteins associated with gene expression, we also find examples where PcG proteins are present at active genes and not present at inactive genes. Our analysis supports the idea that simply the presence of PcG proteins at a target gene does not necessarily result in gene repression and suggests a more dynamic balance between PcG protein binding and gene expression.

## Introduction

As the cells of the embryo progress along developmental pathways they make fate decisions, becoming committed to particular lineages and ultimately to a specific differentiated cell state. Although cell fate decisions may be triggered by transient signals, the resultant cell states are generally stable and are maintained through time and cell division. A long-standing paradigm for understanding the mechanisms underlying the stability of cell fate decisions has been the maintenance of Hox gene expression through gene silencing by Polycomb-group (PcG) genes in *Drosophila* (reviewed in [1]). Hox gene expression domains, initiated in the early embryo through active transcriptional regulation by the transiently-expressed products of the segmentation genes, are thereafter maintained throughout the rest of development and adult life by the maintenance machinery of the PcG and Trithorax-group (TrxG) genes. The products of the PcG genes build the Polycomb Repressive Complexes (PRC1 and PRC2) that are required for gene silencing, whereas the TrxG genes are required for the maintenance of gene activation (reviewed in [2]). In this paradigm, the balance between gene repression and activation is set once and thereafter stably remembered.

A more dynamic view of the role of PcG silencing has recently been emerging, largely from work with embryonic stem cells, where several PcG genes have been shown to be required for both embryonic and adult stem cell maintenance (reviewed in [3]). Genome-wide analysis of the targets of PRC1 and PRC2 complex components reveals that a large number of genes with roles in cell fate decisions and cell differentiation are bound by PcG gene products in stem cells [4],[5]. Many of these genes are repressed by PcG proteins since loss or down-regulation of PcG genes results in their derepression. Upon stem cell differentiation many repressed genes become activated and some concomitantly lose binding of PcG complexes. In stem cells many developmental genes exhibit a “poised” bivalent chromatin organisation, carrying both repressive and active chromatin modifications [6]–[8]. The repressive H3K27me3 histone modification, dependent on the PRC2 complex, is lost from many genes on differentiation. Thus PcG silencing appears to maintain the stem cell state via repression of cytodifferentiation genes; this repression is not permanent and can be lifted upon receipt of differentiation signals.

When the human embryonic teratocarcinoma cell line NT2/D1 is induced to undergo neural differentiation by exposure to retinoic acid, two different scenarios are observed for PcG regulation of target genes [9]. For PcG target genes activated during neuronal differentiation (e.g. the neuronal transcription factor ZIC1 and the neurofilament light chain gene, NEFL), PcG proteins are associated with these genes prior to activation but are lost upon differentiation. In contrast, for PcG target genes repressed during differentiation (e.g. the pro-neural transcription factors OLIG2 and NEUROG2), PcG proteins are already associated with these genes in undifferentiated cells, even though the genes are expressed, and the Polycomb complexes remain after differentiation when expression is switched off. Thus it appears that, at some genes, the association of Polycomb complexes with target genes can change dramatically upon differentiation, but the presence of Polycomb complexes does not always accord with transcriptional repression.

PcG target genes have been identified in *Drosophila* by genome-wide mapping of PcG protein binding in tissue culture cells [10],[11] and by more limited genomic mapping (across 10 Mb of *Drosophila* euchromatin) with different developmental stages in vivo [12]. In this latter study, examples of target genes with clear developmental changes in PcG protein association were identified, suggesting that the chromosome association profile of Polycomb complexes in *Drosophila* may be more dynamic than previously thought.

Here we extend these studies, presenting a genome-wide analysis of PcG proteins in *Drosophila* embryos and in imaginal discs from the third thoracic segment. We examine the binding profiles of Polycomb (Pc), the canonical member of the PRC1 complex, and of Pleiohomeotic (Pho) a DNA-binding protein proposed to recruit the PRC2 complex [13]. Analysis of tissue derived specifically from the third thoracic (T3) segment allows us to examine Pc and Pho association with Hox genes that are known to be either active or inactive in this segment. Comparing binding profiles between the embryo and third larval instar imaginal discs also enables us to examine the dynamics of PcG binding during development of specific tissues. Finally, we compare our in vivo developmental analysis with a previously described genome-wide analysis of Pc binding in *Drosophila* tissue culture cells [10] identifying further examples of differential Pc binding.

## Results

### In Vivo Pc and Pho Target Genes

We performed genome-wide mapping of binding sites for Pc and Pho in chromatin from two in vivo sources; *Drosophila* embryos and imaginal discs. We studied Pc as a representative of the four core PRC1 components, Pc, Polyhomeotic, Posterior sex combs and dRing [14]. We investigated Pho since this is a sequence-specific DNA-binding protein known to be associated with several PcG Response Elements (PREs). Pho binding sites have been shown to be required for PcG-mediated silencing at these PREs [15]–[19] and although Pho is not a component of purified PRC complexes, it interacts biochemically with both PRC1 and PRC2 [13],[20],[21]. Pho co-localises with PRC1 proteins at many sites on polytene chromosomes [16] and, by ChIP (Chromatin Immunoprecipitation) analysis, it is associated with PRC1 binding sites in Hox genes [22],[23].

**Figure 1:**
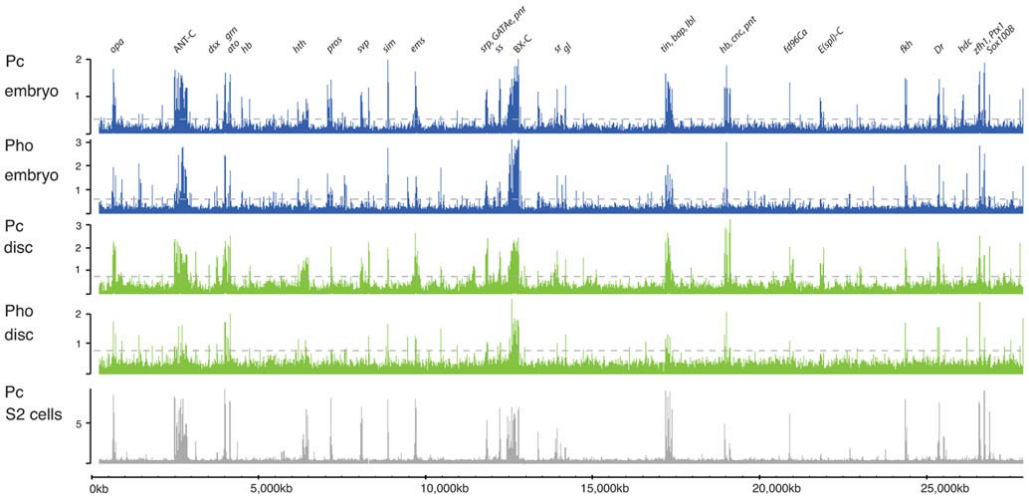
Overview of binding profiles on chromosome 3R. Log_2_ enrichment ratios are plotted for Pc and Pho in embryos and T3 imaginal discs. Threshold enrichment values are indicated by dashed line (3XSD for Pc; 5XSD for Pho) and selected target genes are labelled. The “Pc S2 cells” profile is from Schwartz et al. [10] and plots enrichment ratio.

The 0–16 hr embryonic chromatin provides a base-line for our analysis identifying a set of in vivo targets in a mixture of developmental time-points and tissues. In contrast, the imaginal disc chromatin provides a more focussed sampling of targets within a single tissue (epidermal imaginal), at a particular developmental time (wandering third larval instar) and at a specific position along the body axis (T3 segment). At a gross level, comparison of the binding profiles, e.g. across chromosome 3R as illustrated in Figure 1, reveals considerable similarity, suggesting that Pc and Pho are generally bound at the same locations and that their binding sites appear relatively constant with little change between embryo and imaginal disc.

To analyse these data in more detail, we defined upper and lower binding thresholds for each profile, allowing us to categorise binding over each *Drosophila* gene as positive, intermediate or negative (see Table S1). As well as counting binding events directly over transcription units, intergenic events were separately ascribed to the nearest transcript. Validation of the ChIP-array data by ChIP followed by specific PCR confirmed that thresholds were appropriate (Figure S1).

Using conservative thresholds, we find 386 genes with Pc binding over the transcription unit. 179 (46%) of these are also associated with Pho binding, which rises to 229 (59%) when we include intergenic Pho binding, supporting the idea that Pho has a general role as a DNA-binding protein targeting the assembly of Polycomb complexes.

**Figure 2:**
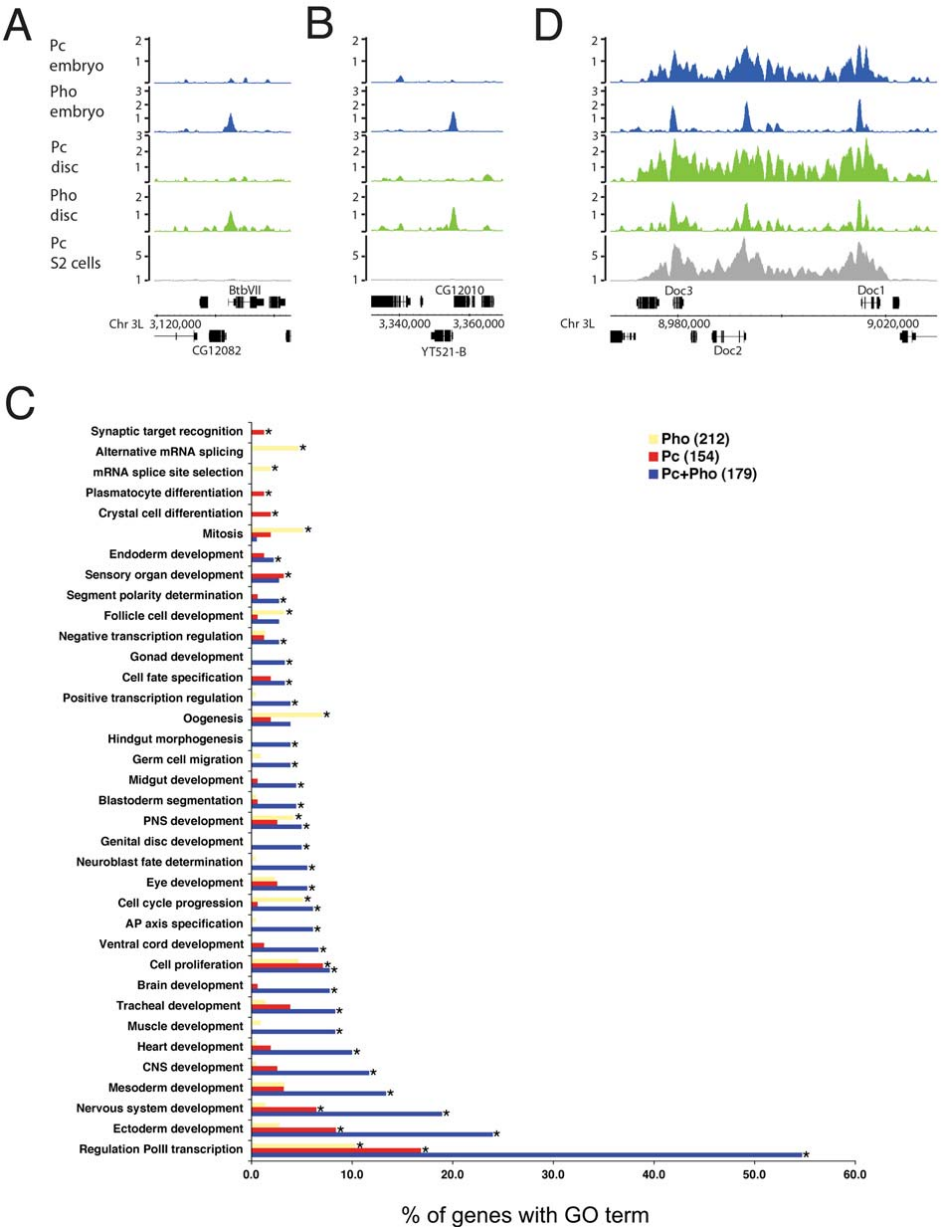
Pc and Pho targets. (A and B) show examples of targets that bind Pho in the absence of significant Pc binding. (C) shows the GO terms associated with specific gene sets derived from the embryo binding data. The “Pho” genes are positive for Pho binding but negative for Pc. The “Pc” genes are positive for Pc binding but negative for Pho. The “Pc+Pho” genes are positive for both. Significantly enriched (corrected p value <0.05) GO terms are indicated with an asterisk. The “Pho” gene set stands out as enriched for certain GO terms including oogenesis, mitosis and alternative mRNA splicing. (D) Enrichment profiles for a selected region comparing the “domain” binding of Pc with the sharper Pho profile.

Interestingly, we find a substantial number of genes (212) that exhibit Pho binding in the absence of Pc (Figure 2). The majority (85%) of these Pho-only binding sites are specific to embryo chromatin. Examination of the GO classifications that are enriched in this subset reveals a markedly different profile from the set of genes that bind both Pc and Pho (Figure 2C). In particular, we find significant overrepresentation (p<0.05) of genes involved in oogenesis, mitosis and mRNA splicing. In addition, we also note that several genes whose products are involved in chromatin regulation (e.g. *brahma*, *Polycomblike, Cp190* and *su(Hw)*) are associated with Pho but not Pc. These observations indicate a role for Pho in the embryo that is independent from its association with Pc.

### Binding Site Analysis

As illustrated in Figure 2D the local profiles of Pc and Pho binding are very different. Pc is often associated with a broad binding domain extending over tens of kilobases whereas Pho binding is characterised by much narrower isolated peaks. Since the sharp Pho peaks identify relatively short regions associated with Pho binding we searched for enriched sequence motifs underlying these peaks. In addition to Pho, several other DNA-binding proteins, including GAGA factor and Zeste, are associated with PREs [24]–[29]. We were interested to see if Pho-bound sequences exhibited the canonical Pho binding motif, as well as putative binding sites for these other factors.

**Figure 3:**
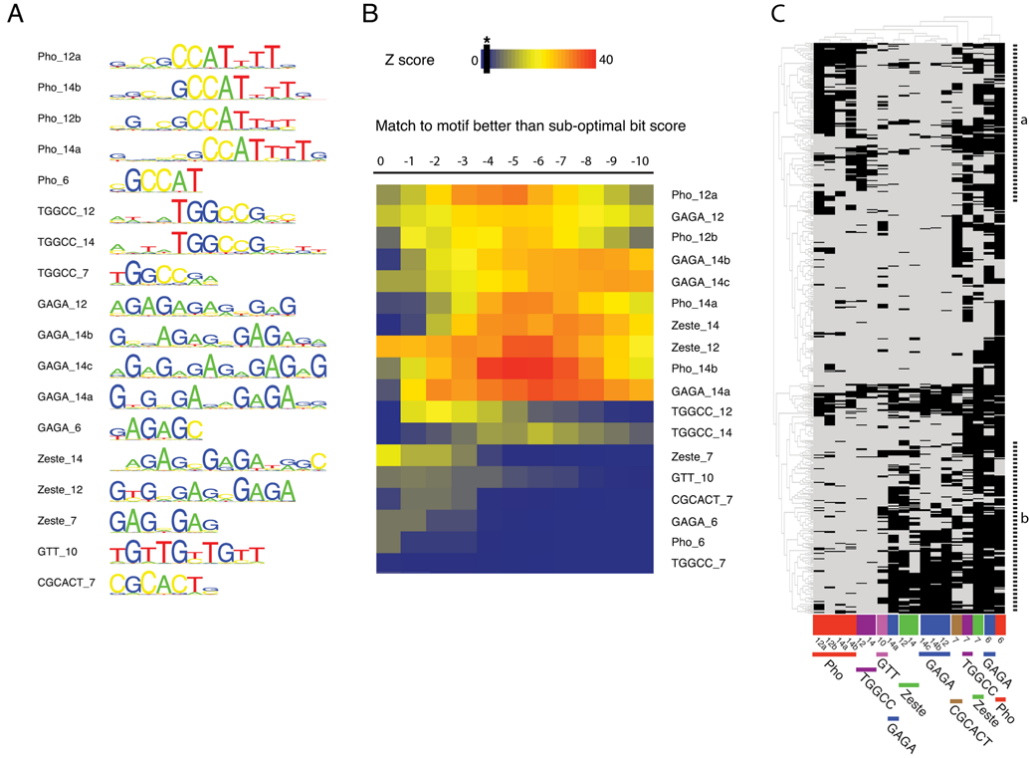
Sequence motifs identified in Pho-bound regions. (A) Selection of 18 motifs chosen for further analysis. The motifs can largely be grouped into clusters of similarity to the canonical Pho, GAGA and Zeste binding sites. The TGGCC motifs form a distinct class related to the canonical Pho motif. The GAGA- and Zeste-like motifs differ as Zeste-like motifs have a pronounced “GAG”, although this classification is arbitrary. Motifs are named according to class with a suffix indicating motif length. (B) Motif over-representation heat map. For each motif, a search for sequence matches with decreasing similarity (sub-optimal bit score) was conducted. The over-representation is measured as a Z-score, comparing the number of actual Pho peaks containing the motif to the number of occurrences in a random data set. This analysis confirms the statistical over-representation of all motifs, and suggests cutoff values for the sub-optimal bit score. (C) Bi-clustering of motif occurrence in 628 embryonic Pho peaks. Each column represents one of the 18 selected motifs, each line one of 628 embryonic Pho peaks. Rows and columns are ordered according to similarity. Motif presence better than the motif-specific sub-optimal bit score is denoted as a black mark. The motif type is indicated below the diagram. Peaks in region indicated by (a) have long Pho/TGGCC motifs but lack long GAGA/Zeste motifs while the peaks in region (b) show the opposite.

We learned motif dictionaries from the central areas of 150 strong Pc-associated Pho peaks using various search parameters. The searches identified more than 70 partially redundant sequence motifs and we selected 18 of these for further analysis, based on their length, information content and/or similarity to known binding sites of Pho, GAGA and Zeste (Figure 3, see also Table S2 and Dataset S1). Binding sites of additional factors known to be involved in Pc recruitment (e.g. Grainy head) could not be identified. We found Pho-type motifs comprising a core GCCAT sequence with a more or less pronounced tail of four Ts. In addition, we found a novel TGGCC motif that may be related to the Pho-type as it has a GCC core (and GCCA on the reverse strand) but which lacks the tail of Ts. We also found a frequently occurring GTT repeat and a CGCACT sequence motif. The GAGA- and Zeste-type motifs differ in that Zeste-like motifs have a pronounced “GAG”, however we recognise that this classification is somewhat arbitrary.

The selected motifs were significantly over-represented when we compared their occurrence in all Pho peaks to random sets of sequences that were not occupied by Pho (Figure 3B). While longer motifs with positions of low information content are mostly over-represented when allowing for mismatches, short sequence motifs are only over-represented when considering perfect matches or small sub-optimal bit scores. This over-representation approach also enabled us to derive informed cutoff values for further analyses; for each motif we identified the sub-optimal bit score for which the motif shows the strongest evidence for over-representation. Using these cutoff values, we determined the presence of the different motif types in all 628 embryonic Pho peaks (Figure 3C). The short motifs (clustered to the right of the diagram) are well represented in the Pho peak sequences; 85% of the sequences contain Pho_6, 63% contain GAGA_6 and 64% contain Zeste_7. Longer Pho and TGGCC motifs occur in 51% of the peaks and have an interesting antagonistic clustering to the longer GAGA and Zeste-type motifs; i.e. peaks containing longer Pho/TGGCC-type motifs (region A in Figure 3C) cluster separately from peaks containing longer GAGA/Zeste-type (region B). Overall, the general association of Pho, GAGA and Zeste binding motifs with Pho binding is consistent with the clustering of these motifs previously used to predict PREs in the *Drosophila* genome [29] and we add novel enriched sequences that may improve such approaches. However, we emphasise that there is considerable variability in the motif occurrence at Pho peaks as we illustrate for representative peaks in Figure S2.

We were interested to determine if there was any qualitative difference in motif composition between Pc-associated and Pho-only peaks. We compared the number of peaks containing particular motifs for both these peak classes and tested for significance using chi-square statistics. Interestingly, we find significant differences with TGGCC_7 (34.7% vs 53.6%, p<3×10^−6^) and Zeste_7 (31% vs 46.8%, p<8×10^−5^) under-represented in the Pho-peaks that are not associated with Pc. In contrast, the Pho_12a (43.9% vs 26.2%, p<3×10^−6^) and Pho_14b (35.2% vs 20.4%, p<3×10^−5^) motifs are over-represented in the Pho-only group. This observation highlights the potential importance of a long Pho motif at the Pho-only sites. There is no compositional bias for the longer GAGA- and Zeste-type motifs.

### Pc and Pho Are Bound at Both Inactive and Active Hox Genes

What differentiates a silenced from an active Hox gene? Although the PcG and TrxG genes have antagonistic effects on gene expression they can nevertheless be present at the same gene. PcG proteins and TrxG proteins were found to co-localise at targets on salivary gland polytene chromosomes [30],[31] and at PREs in the Bithorax Complex (BX-C) [32]. In addition, Pc-binding does not correlate with gene expression in S2 cells [33]. Recently, Papp and Mueller have analysed the binding of PcG and TrxG at the *Ubx* gene in active and repressed states in vivo [22]. Sampling the occupancy of these complexes at 17 sites across 115 kb of the *Ubx* region, encompassing the transcription unit and regulatory sequences, they found that PcG proteins of both the PRC1 and PRC2 complexes as well as Trx protein are bound to *Ubx* PREs in both the ON and the OFF states. Similarly, Trx and PcG were found to co-localise at binding sites in both active and inactive Hox genes in tissue-culture cells [23].

Our ChIP-array data allows a more extensive assessment of Pc and Pho occupancy across the *Drosophila* Hox complexes in vivo. Chromatin derived from embryos represents a mix of active and inactive states for the different Hox genes, however, for the T3 imaginal discs we can compare silenced and active Hox genes. Focusing on the BX-C: *Ubx* is active in the T3 discs, where it is required in the haltere disc to specify haltere in contrast to wing development, and in the T3 leg disc to specify T3 characteristics. In contrast, both *abdominal-A* (*abd-A)* and *Abdominal-B* (*Abd-B)* are silenced in T3 discs.

**Figure 4:**
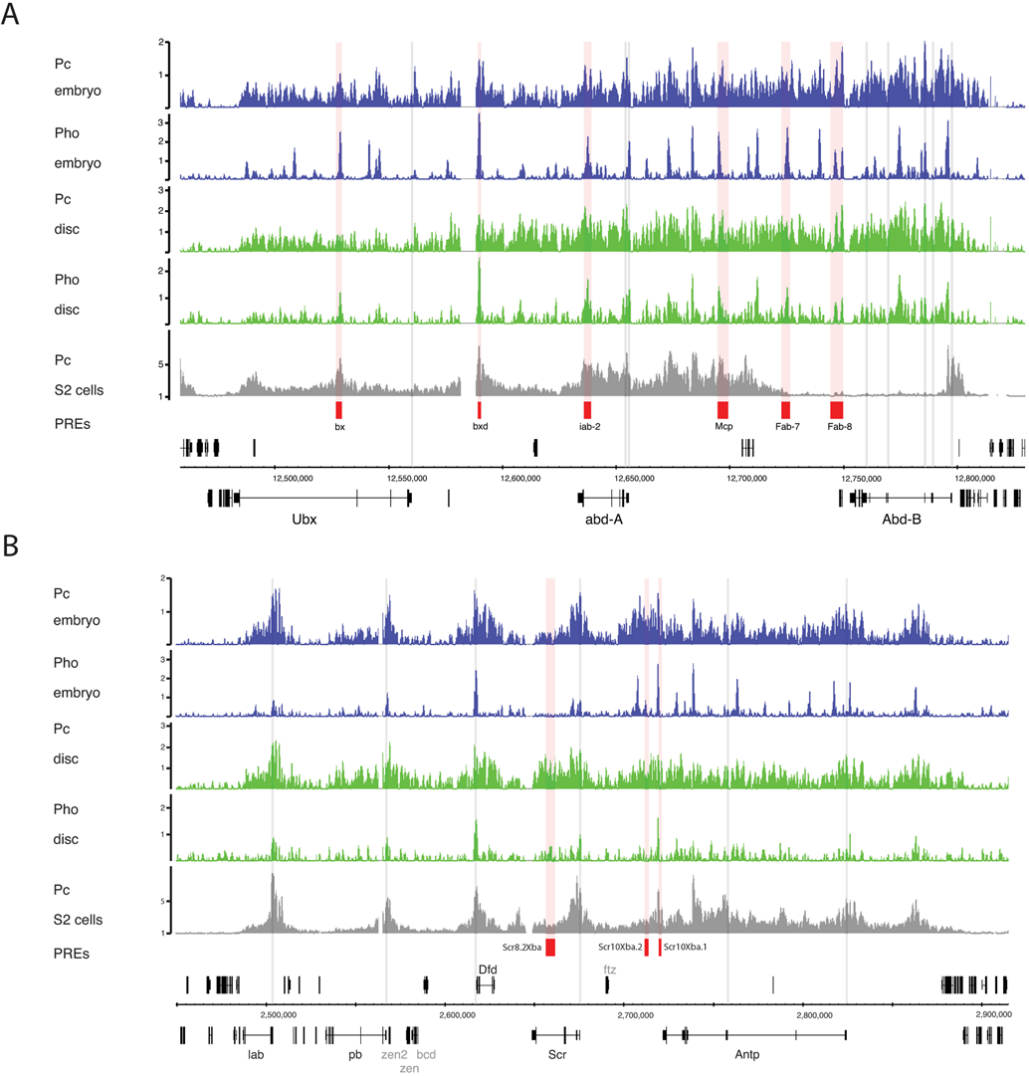
Pc and Pho binding profiles at the Hox complexes. Enrichment profiles across the BX-C (A) and the ANT-C (B) showing broad Pc binding and more discrete Pho peaks. Characterised PREs are shown in red and Hox gene promoters as grey verticals. (A) In the BX-C characterised PREs are generally marked by Pho peaks and maximae in the Pc profile but there are many more Pho peaks than characterised PREs. Significant Pc and Pho binding is associated with the three BX-C Hox genes in both the embryo and in the T3 discs despite the active expression of *Ubx* in the T3 discs. (B) In the ANT-C there is a strong correspondence between Pho peaks/Pc maximae and the Hox gene promoters particularly in the cases of *lab, pb, Dfd* and *Scr*. The long *Antp* transcription unit is associated with multiple Pho peaks. One of the previously characterised PREs is associated with a major Pho peak. Significant Pc and Pho binding is associated with the ANT-C Hox genes in both the embryo and in the T3 discs even though *Antp* is not transcriptionally silenced in the T3 discs. Note the other homeobox genes in the region, *zen, zen2, bcd* and *ftz* are not associated with prominent Pc or Pho binding (genes shown in grey).

The Pc binding profile shows an extensive domain of binding that covers the approximately 300 kb BX-C region (Figure 4A). Characterised PREs tend to be represented as regions of relatively higher binding within the overall domain. As with other regions of the genome, the Pho binding profile is very different with much sharper, more isolated peaks several of which coincide with characterised PREs. In chromatin from the T3 imaginal discs both Pc and Pho are associated, as expected, with the silenced genes, *abd-A* and *Abd-B*. However, we also find significant association with the active *Ubx* gene. The T3 disc Pc profile over *Ubx* is similar to the embryo chromatin profile with an extensive domain and significant binding at both the *bx* and *bxd* PREs as well as a peak close to the start of *Ubx* transcription. The Pho profile in T3 discs also shows clear peaks at these PREs and binding close to the *Ubx* 5′ end. These data show clear evidence of Pc and Pho association with an active gene and confirm and extend the results of Papp and Mueller [22].

There are, however, differences between the embryo and T3 disc profiles. For example, several strong Pho binding peaks in embryo chromatin are only weakly represented in the T3 disc chromatin. In addition, there is a generally lower level of Pc and Pho binding across the active *Ubx* gene in comparison to the inactive *abd-A* and *Abd-B* genes. The average enrichments (log_2_ binding ratios) across the three transcription units in disc chromatin for Pc are: *Ubx* 0.33, *abd-A* 1.03 and *Abd-B* 1.01 and in the case of Pho: *Ubx* 0.07, *abd-A* 0.31 and *Abd-B* 0.34.

Significant Pc and Pho binding associated with an active Hox gene is also found in the *Antennapedia* Hox cluster (ANT-C). This cluster contains the Hox genes *lab, pb, Dfd, Scr* and *Antp,* and all these genes are associated with widespread Pc binding and distinct Pho peaks in the embryo (Figure 4B). It is striking that peaks in the Pc distribution as well as strong Pho peaks are found close to the 5′-ends of *lab, pb, Dfd* and *Scr*. The longer *Antp* gene is covered by a domain of Pc binding and is associated with several Pho peaks. As with *Ubx*, *Antp* is active in T3 discs since it is expressed from the labial segment posteriorly. Indeed, *Antp* may be a better gene than *Ubx* for the analysis of the active state in T3 because it is expressed in both the ectoderm and mesoderm of the T3 segment [34],[35], whereas *Ubx* is only active in the ectodermal imaginal cells of the T3 disc and may be silenced in the small population of mesodermal adepithelial cells [36]. Despite this difference we find a similar situation with *Antp* as we observe with *Ubx*. Although it is active, *Antp* is nevertheless associated with a significant domain of Pc binding, which encompasses the *Antp* transcription unit, as well as strong peaks of Pho binding close to the 5′ and 3′ ends of the gene. Other Pho peaks over *Antp* that are prominent in embryo chromatin are less apparent in T3 discs.

As shown in Figure 4 we find many more Pho binding peaks across the Hox complexes than there are characterised PREs. As all these Pho sites may not be functionally equivalent we examined the motif composition in the 36 Pho peaks in the BX-C. We find a high variability of motif counts but characterised PREs do not appear as a distinct motif-rich group (Figure S3).

### Tissue-Specific Differences in Pho and Pc Binding

While our analysis of the Hox clusters demonstrates significant Pc and Pho binding associated with both active and inactive genes, a genome-wide comparison of the embryo and T3 imaginal disc profiles shows that PcG proteins are not constitutively associated with target genes. The binding profiles of embryo and T3 disc chromatin are similar, with 65% (252/386) of the genes significantly associated with Pc in the embryo also above threshold in the disc chromatin. This rises to 81% (314/386) if we include the genes with intermediate binding in discs. When we include the data from the genome-wide S2 tissue culture study [10] we also find considerable overlap. For genes with direct binding over transcription units there is a 42% (161/386) overlap across all three data sets rising to 58% (224/386) if we include intermediate binding in discs and S2 cells. A gene-by-gene comparison is provided in Table S1.

Comparison with other previously published datasets also reveals considerable overlap. For the analysis of Pc targets by the DamID method in Kc tissue culture cells [11] we find that our set of Pc targets in the embryo (386 targets) contains 136 targets from the Kc cell data. As the genome coverage of the Kc cell analysis is approximately 60%, this extrapolates to an estimated 60% overlap. This comparison is detailed in Table S1. There is also good correspondence with the in vivo data from Negre et al. [12] where, for example, 5 out of the 7 targets they identify in the 3 Mb *Adh* region in embryo chromatin are also present in our set of Pc targets in the embryo. A detailed comparison is presented in Figure S4.

For examining differential Pc binding, we focus on the most comparable datasets, the two chromatin samples in our dataset and the Schwartz et al. S2 data [10], as these three datasets are genome-wide and use the same Affymetrix array platform. Despite the overall similarity comparing the embryo, T3 disc and S2 cell chromatin samples, there are clear differences in Pc occupancy for some genes indicating potential sites of differential Pc activity. To reduce the level of artificially selected differential Pc targets resulting from automatic selection of peaks in the high-throughput analysis, we visually screened the binding profiles of all differentially bound regions and restricted our further analysis to differential gene sets that show significant enrichment of GO categories. We identify three robust sets of differentially occupied genes. We find 49 genes that are bound by Pc in the embryo but are not Pc-associated in imaginal discs, 107 genes that are bound by Pc in the embryo but not in S2 cells and 119 genes that are bound by Pc in imaginal disc but not in S2 cells.

**Figure 5:**
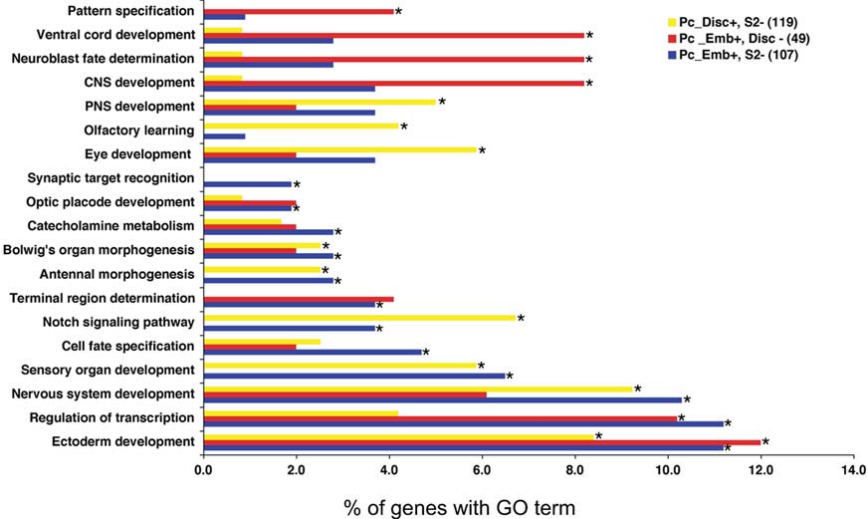
GO analysis on differentially bound Pc targets. “Pc_Disc+, S2-“ are positive for Pc binding in T3 discs but negative in S2 cells. “Pc_Emb+, Disc –“ are positive for Pc binding in embryos but negative in T3 imaginal discs. “Pc_emb+, S2-“ are positive for Pc binding in embryos but negative in S2 cells. Significantly enriched GO terms (corrected p value <0.05) are indicated with an asterisk. The GO class “regulation of transcription” is plotted; the related GO term “regulation of transcription from RNA polymerase II promoter” is also relevant with 33% of genes with this term in “Pc_Emb+, Disc –“ and 21% in “Pc_emb+, S2-“. The Pc targets that are differentially unoccupied in S2 cells are associated with ectoderm/neural differentiation and the genes selectively unoccupied in T3 discs are associated with early CNS development.

By examining the genes in these differentially-bound sets we anticipated that we might identify cell fate-determining genes for cell fate decisions in particular developmental pathways. For example, genes bound by Pc in the embryo but not in imaginal discs might represent genes released from Pc silencing on the pathway of disc development and hence might represent key cell-fate determining genes for that pathway. However, the GO analysis of these gene sets (summarised in Figure 5 (also see Table S3)) presents a striking observation. The Pc target genes that are unoccupied by Pc in a particular tissue appear to have little to do with cell fate decisions that are relevant for that tissue. For example, the genes occupied by Pc in the embryo but not in the imaginal discs are enriched in genes involved in fate decisions in neuroblasts of the central nervous system. Similarly the genes that are occupied in embryos but not in S2 cells, which are mesodermally–derived cells of the haemocyte lineage, are genes required for ectodermal and neural fate decisions. The genes that are occupied in imaginal discs but not in S2 cells are relevant for fate decisions occurring in discs but not in S2 cells (e.g. sensory organ development, eye development, ectoderm development).

**Figure 6:**
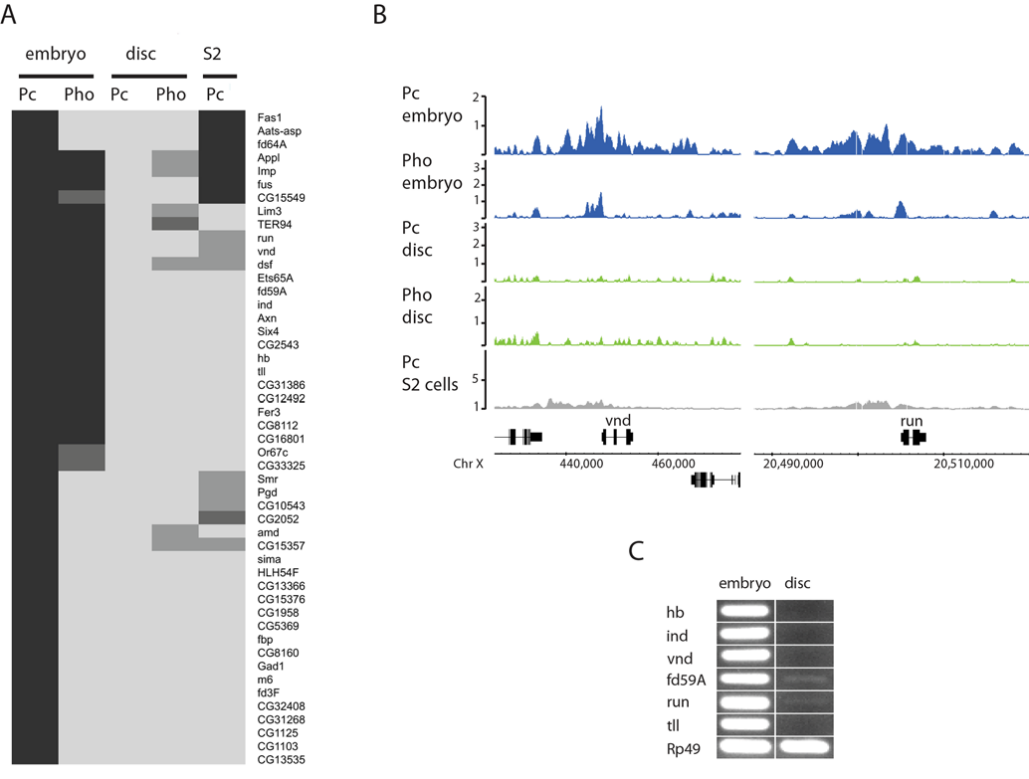
Pc targets bound in the embryo but not in T3 imaginal discs. (A) Cluster diagram showing binding classification for the 49 genes positive for Pc binding in embryo but not in T3 discs. Black: positive peak overlapping transcription unit; dark grey: positive peak closer to this gene than any other gene; mid grey: intermediate peak overlapping transcription unit; pale: negative. The Pho binding follows the Pc binding and the 49 genes are also generally negative for Pc binding in S2 cells. (B) Example binding profiles for genes in this class showing *vnd* and *run*. (C) RT-PCR analysis on selected genes shows expression in embryos but little or no expression in T3 discs. Rp49 provides a positive control.

We further investigated the group of 49 genes which are associated with Pc in the embryo but not in T3 discs. Figure 6A lists these genes and shows their pattern of binding of Pc and Pho across the five data sets. Representative binding profiles are shown in Figure 6B.

As the plot in Figure 6A demonstrates, approximately half of the Pc targets also show Pho binding in the embryo (49%) and, as with Pc, Pho binding is absent in the T3 discs. For this gene set, target occupancy in the S2 cells is similar to that observed in imaginal discs with only a few targets (14%) showing Pc binding. The set of 49 genes specifically bound in the embryo contains several well-characterised genes; notably *run, hb* and *tll* that are involved in early embryonic patterning and in neuroblast specification as well as two genes, *ind* and *vnd* involved in the specification of the nervous system and in neuroblast fate. As mentioned above, this class of genes has a strong GO signature and is highly enriched in transcription factors (Figure 5). Some individual classes of transcription factor are particularly strongly enriched. For example, 3 out of the 19 forkhead domain proteins present in the *Drosophila* genome are represented (hypergeometric probability 3.5E-05) and 3 putative hormone-receptor C4-zinc finger genes of the 21 in the genome (4.7E-05).

The GO analysis and the individual gene functions suggest that this set of genes may be involved in early embryonic fate decisions but not in fate decisions that are relevant for the imaginal disc cells, where these targets are unoccupied. To explore this we asked whether these genes are actually expressed in imaginal discs. All of the six genes tested for expression by RT-PCR are expressed the embryo but show little or no expression in imaginal discs (Figure 6C). Thus, as with the Hox genes, we find Pc occupancy is not linked to expression state in a simple fashion.

### Pc and Cell Fate Genes in the Haemocyte Lineage

*Drosophila* S2 cells are an embryo-derived cell line that appear to be related to haemocytes since they are phagocytic and express haemocyte markers [37]. We were interested to relate the Pc binding profile in these cells to the genes involved in cell fate decisions in the haemocyte lineage (reviewed in [38]). The embryonic haemocytes are derived from a head mesoderm primordium defined by the GATA transcription factor, Serpent (Srp), and differentiate into crystal cells or plasmatocytes. Lozenge (Lz), a Runx family transcription factor, is required for crystal cell development whereas U-shaped (Ush) antagonises crystal cell development and Glial cells missing (Gcm) promotes plasmatocyte development. The closely related Gcm2 acts redundantly with Gcm in plasmatocyte differentiation. Full maturation of plasmatocytes requires the PDGF/VEGF Receptor (Pvr). S2 cells express *srp* together with the plasmatocyte markers *ush* and *pvr* and do not express the crystal cell marker *lz* (FLIGHT database: http://flight.licr.org/, [37]). The expression status of *gcm* and *gcm2* is less clear; they are not scored as expressed in S2 cells in the FLIGHT database but are reported to be detectable by RT-PCR [37].

**Figure 7:**
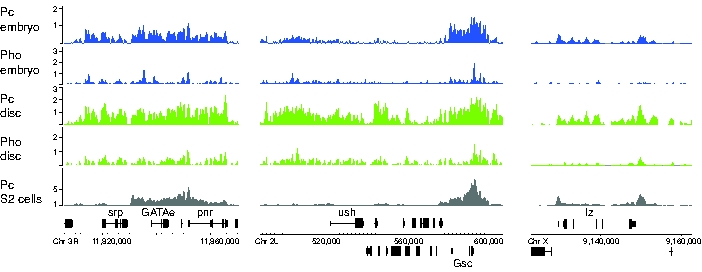
Binding profiles for haemocyte lineage genes. The *srp* and *ush* genes expressed in S2 cells show Pc and Pho binding in embryos and imaginal disc but do not show Pc binding in S2 cells. Nearby genes provide positive controls (e.g. *Gsc* in the case of *ush*) and note the sharp boundary between Pc binding at *srp* and the neighbouring *GATAe* gene. The *lz* gene is not expressed in S2 cells and is associated with Pc binding.

The key cell fate-determining genes in the haemocyte lineage, *srp*, *lz*, *gcm*, *gcm2* and *ush*, are all Pc targets. Figure 7 compares the Pc and Pho occupancy at *srp, ush* and *lz* in S2 cells with the occupancy in embryos and imaginal discs. Strikingly, the cell fate genes associated with the plasmatocyte fate, *srp* and *ush*, show strongly reduced Pc occupancy in S2 cells compared to embryos and imaginal discs whereas the crystal cell determining gene, *lz*, shows clear Pc binding. The comparative binding at *srp* is dramatic as there is a highly specific reduction in Pc binding in a specific domain over the *srp* gene in S2 cells, whereas the neighbouring gene *GATAe* is strongly associated with Pc binding.

Overall, this analysis of Pc binding at cell fate-determining genes in the haematocyte lineage shows clear differential binding in S2 cells that correlates with gene expression and the requirement for gene activity in the plasmatocyte pathway.

### Pc Target Gene Expression and Specific Cell Fates

**Figure 8:**
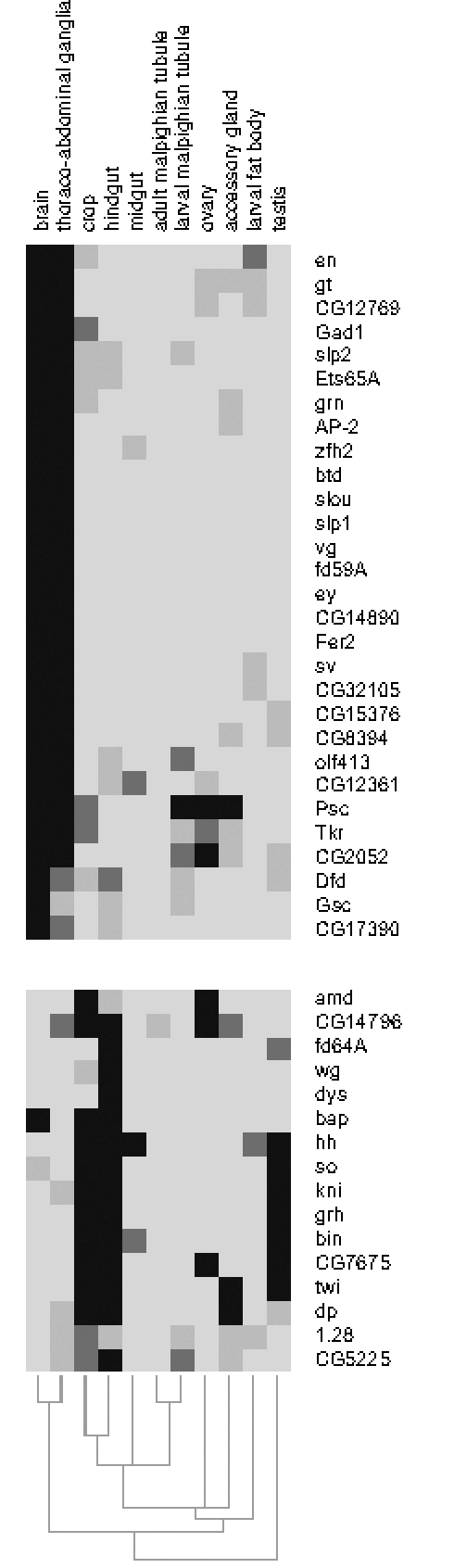
Tissue expression of Pc targets. Selected regions from the cluster analysis of 373 Pc target genes clustered according to expression in larval/adult tissues using the FlyAtlas expression data. Intensity represents the number of present calls in replicates (maximum 4). Related tissues cluster together and the selected regions show gene clusters for brain/thoraco-abdominal ganglia and crop/hindgut. Many genes relevant for early developmental decisions in these tissues are still expressed in the adult. The full cluster diagram for the 373 Pc target genes is given in Figure S5.

The Pc maintenance machinery functions to stably propagate states of gene activity through cell division and, for the Hox genes, stable expression patterns are preserved throughout development. We were interested to examine if this is also true for other Pc target genes. If Pc targets in general are stably expressed once activated, then differentiated cells may express the set of Pc target genes that have been activated along the developmental pathway they have followed. We used the FlyAtlas data set (http://www.flyatlas.org/) of gene expression profiles from selected adult and larval tissues to examine the pattern of deployment of Pc target genes in specific tissues [39]. Out of the 386 Pc target genes we identified with embryo chromatin, we obtained tissue-specific expression data for 373 genes from FlyAtlas. The cluster analysis of the expression data is presented in Figure 8 (and Figure S5). We find that the data for this small sample of genes out of the 18,770 transcripts in the data set nevertheless clusters according to tissue type. For example, the two neural samples, brain and thoracic/abdominal ganglia cluster together, as do the crop and hindgut samples representing ectodermal-derived gut ensheathed in visceral mesoderm. Thus the Pc target gene expression profile provides a tissue-type signature. The cluster diagram, in addition to the block of genes that are present in all samples, also provides several blocks of genes whose expression is related to particular tissues. For example, the block illustrated in Figure 8B includes genes expressed in foregut (crop) and in hindgut and contains the two key genes *bin* and *bap* that are required for the specification of the visceral mesoderm in mid-embryogenesis. This analysis indicates that not only the Hox genes but also other Pc target genes remain stably expressed through to adulthood, providing a basis for the stable specification of cell type.

## Discussion

The PcG target genes identified by several genome-wide binding studies represent an assembly of key regulators that generate cellular diversity and patterning in the developing organism [4],[5],[10]. Coupled with the ability of the PcG/TrxG maintenance machinery to stably transmit states of gene expression through cell division, this provides a system for the stable inheritance of cell fate decisions and for the stability of differentiated cell states. In this paper we have examined the linkage between the PcG machinery and cell fate decisions by comparing the binding sites of PcG machinery components in different tissues. We have generated genomic binding profiles for Pc and Pho from whole *Drosophila* embryos and from the specific tissue of the imaginal discs of the third thoracic segment. We combined our data from in vivo sources with the data from *Drosophila* S2 tissue culture cells [10] allowing comparison of PcG occupancy in chromatin from different tissues. In general we find considerable overlap of target sites in the three data sets. However Pc and Pho binding to target sites is not simply constitutive and we find clear examples of alterations in Pho and Pc binding at specific target sites in particular tissues.

We find a substantial number of genes associated with Pho binding but not Pc, suggesting a function for Pho separate from its role in PcG-mediated gene silencing. Pho has been found to be associated with two distinct protein complexes, Pho-dINO80 and PhoRC [40]. PhoRC is implicated in PcG-mediated gene silencing and it contains dSfmbt, a PcG protein required for Hox gene repression. PhoRC, but not the Pho-dINO80 complex is bound at PREs. The role of the Pho-dINO80 complex is unknown but it is a candidate for mediating Pho function at the Pc-independent Pho targets. Null *pho* mutants lacking any maternal contribution exhibit severe pleiotropic phenotypes and one *pho* allele shows a specific female sterility phenotype [15]. In this respect it is interesting to note that the Pho target genes we identify are overrepresented for oogenesis, mitosis and splicing functions. Of the 212 Pho-only targets, 60% are enriched for ovary expression and 66% are absent in testis according to FlyAtlas (http://www.flyatlas.org/). This suggests that *pho* may regulate a set of specific functions during oogenesis and we suggest that Pho continues to be associated with these targets in the embryo. A more general role for Pho, separate from PhoRC function, is also suggested by clonal analysis of *pho* and *dSfmbt* mutations in imaginal discs [40]. Mutant clones lacking *pho* (together with *pleiohomeotic like* which functions largely redundantly with *pho*) show not only loss of Hox gene silencing but also growth defects that result in the elimination of the clones from the disc epithelium. Clones lacking dSfmbt lose Hox silencing but do not show growth defects. Recently, Pho was found to be bound at active genes and is strongly recruited at sites with high transcription (chromosome puffs) on salivary gland polytene chromosomes. Based on the kinetics of Pho binding at heat-shock loci a role for Pho in the repression of previously active genes was proposed [23].

Examination of Pc and Pho binding in the BX-C in T3 imaginal discs provides a clear test case for the linkage between occupancy and gene expression since *Ubx* is expressed but *abd-A* and *Abd-B* are silenced. We find significant Pc and Pho binding associated with both the expressed and the silenced genes. This provides a clear demonstration that silencing is not simply established by the presence of PcG proteins at a target site and supports previous observations of a lack of correlation between PcG binding and gene silencing [22],[23],[33]. Although the *Ubx* gene is associated with significant Pc and Pho binding, there is overall less binding over *Ubx* in comparison with the two silenced genes. Also, the Pho binding profile in the embryo, representing a mixture of gene activity states, is markedly different from the T3 disc profile; the T3 profile shows prominent peaks at the *bx* and *bxd* PREs but the other peaks seen with embryo chromatin are less prominent. Similar effects are also seen at the *Antp* gene, which is also active in T3 discs. Reduction and rearrangement, rather than absence, of PcG protein at active genes suggests a dynamic interaction between silencing and gene transcription. Indeed, Pc complexes have been shown to be highly dynamic with rapid exchange of PcG proteins on chromatin [41]. Alteration of the Pho binding profile at Hox genes in cell lines with differential Hox expression has also been reported [23] with the striking observation of spreading of the Pho binding at active loci. Such dramatic Pho spreading is, however, not apparent in our data.

We identified a set of 49 Pc target genes that were bound by Pc in the embryo but not in the T3 imaginal discs. We had expected that such a class might contain genes required for fate decisions on the pathway to T3 imaginal disc cell differentiation and were surprised to find that this gene set was enriched in genes required for early cell fate decisions in the nervous system. We examined the expression of several of these genes and found little or no expression in the T3 discs, reinforcing the idea that Pc binding does not simply correlate with silencing of gene expression. In the case of *Ubx* and *Antp* we find that expressed genes have significant Pc binding and, with the set of genes that show Pc association in embryos but not in imaginal discs, we find inactive genes that lack Pc. This is reminiscent of an observation with the Pc target *hedgehog* (*hh*) which has an identified PRE and is silenced by PcG in the embryo and imaginal discs [42],[43]; in salivary gland polytene chromosomes, Chanas and Maschat found no PcG binding at the *hh* gene despite the fact that *hh* is not expressed in this tissue [43]. A similar observation was made for CycA [44]. In this case we note that although *hh* is a clear Pc target in S2 cells and in our in vivo analysis, CycA is not [10]. In all of these cases of differential Pc binding it is possible that the particular genes are inactive due to the absence of appropriate transcription factors to drive expression in particular tissues. This contrasts with the situation in the Hox genes where Pc is continuously required to maintain silencing against a background presence of gene activators [45]–[47]. If Pc complexes are only recruited to genes where they are required to counteract gene activation, this would provide an economical way to deploy the silencing machinery. It would also imply the existence of a mechanism that enables the PcG-machinery to identify genes that are capable of being activated. A possible basis for this mechanism could be the targeting of Polycomb complexes by non-coding RNAs; PcG proteins are recruited by non-coding RNAs in mammalian X chromosome inactivation [48] and recent studies implicate non-coding RNA in Hox gene repression [49],[50]. Alternatively, the lack of Pc associated with non-expressed genes may indicate that these genes are repressed through non-PcG dependent mechanisms. In the case of *hh* in the salivary glands, there is some support for this since attempts to activate *hh* by supplying activators were unsuccessful [43]. In addition, a study on histone modifications and cell lineage provided evidence for a class of genes that lose both the PcG-dependent H3K27me3 mark associated with silencing and the TrxG–dependent H3K4me3 mark associated with activation on lineage progression [8]. Loss of both marks was found to be associated with gene inactivity.

In the case of the haemocyte lineage cell fate-determining genes required for plasmatocyte development, these genes are expressed in S2 cells and are found to be selectively unoccupied by Pc. This is what would be expected for a non-silenced active gene and fits with the idea that Pc is lost from PRE/TREs following switching to the active state. However it raises the question of why *Ubx* or *Antp*, as genes expressed in T3 imaginal disc cells, are still associated with significant Pc binding whereas a Pc target gene such as *srp* appears to lack Pc binding in haemocyte lineage cells. S2 cells are tissue culture cells whose gene regulatory systems may have deviated considerably from the endogenous state and it is therefore possible that the observed Pc status represents a tissue culture artefact. However, another possibility is that it relates to plasticity. Imaginal disc cells are relatively undifferentiated precursor cells that only differentiate fully during metamorphosis. S2 cells, on the other hand, may represent a more committed cell state. In this respect it is interesting to compare the Pc profiles observed at the BX-C in S2 cells and T3 imaginal discs (Figure 4A). S2 cells express high levels of *Abd-B* but very low levels of *Ubx* and *abd-A*. Pc binding reflects this gene expression and in particular shows no binding over an *Abd-B* domain that includes the four active *Abd-B* promoters [10]. This contrasts with the situation in T3 imaginal discs where the active *Ubx* gene is associated with significant Pc binding. However, it should be noted that the state of Hox gene expression in S2 cells is rather curious since these cells are thought to derive from the head mesoderm, an area of the embryo that does not express any of the genes of the BX-C. Despite this caveat, the differences in the Pc binding at active genes in S2 versus imaginal disc cells may reflect the plasticity of the undifferentiated imaginal disc cells compared to the loss of plasticity in the S2 cells. In general, we have identified two situations where Pc target genes are not bound by Pc proteins, a set of inactive genes in imaginal discs and a set of active genes in S2 cells and the common feature may be that these both represent terminal stable gene states. In these situations, loss of Pc binding may be associated with loss of plasticity and may indicate final cell commitment.

Our analysis of T3 imaginal discs enabled us to investigate the Pc occupancy of genes in this specific tissue but does not immediately reveal the developmental history of these cells in terms of which cell fate switches had been turned on and which had been turned off along the developmental pathway leading to T3 imaginal epidermal specification. The Pc target genes which exhibited no Pc binding in the T3 imaginal discs did not obviously suggest a set of fate-determining genes for T3 disc specification. In the relatively undifferentiated imaginal disc cells it is apparent that Pc occupancy by itself does not differentiate a silenced from an active state and so to map the fate switching history of a cell we will either need to find markers that provide a clearer readout of the state of gene activation or else we will have to look at more differentiated cells where the PcG system has stabilised. Our analysis of a limited set of adult tissues, where gene expression data is available, provides support for stable activation of cell fate decision genes, suggesting that examining the expression of Pc target genes in differentiated cell types can provide information on the key developmental genes that are activated on a specific developmental pathway. Although the T3 imaginal discs represent a tissue sample of limited cell fate diversity they are nevertheless a complex mixture of cells with different states of gene activity. Many of the known key genes in imaginal disc development e.g. *vg, Dll, hh* and *tsh* are active in only a subset of disc cells and therefore the Pc and Pho binding profiles we observe may represent a mixture of active and silenced states. Further analysis examining more restricted tissues will be required to investigate to what extent the Pc target genes provide a stable “genetic address” [51] specifying cell differentiation.

## Materials and Methods

### Fly Stocks and Antibodies

The wild type strain used was OregonR. The Pc-GFP transgenic fly line was generated by Dietzel et al. [52]. The antibodies used were affinity purified rabbit anti-GFP [53], rabbit anti-Pho [18] and affinity-purified anti-Pc [54].

### Chromatin Isolation and Immunopurification

Chromatin from embryos aged between 0 to 16 h after egg laying was purified as described previously [55]. For the preparation of chromatin from T3 imaginal discs (haltere and third leg) late 3rd instar larvae were dissected in ice-cold Schneider's Medium. Dissected discs were washed with PBS, fixed in PBS/1.5% formaldehyde for 20 min and washed with PBS. Batches of material were snap-frozen in liquid N_2_ and stored at −80°C. Chromatin was prepared from a minimum of 100 discs. For Pc target analysis the specific reaction used chromatin from the Pc-GFP fly line immunopurified using anti-GFP, and the control reaction used wild type chromatin immunopurified using anti-GFP. The Pc-GFP protein binds to the same polytene chromosome loci as wild-type Pc [41] and we validated a selection of targets by ChIP using anti-Pc antiserum (Figure S1). For Pho analysis wild type chromatin was used with anti-Pho for the specific reaction and pre-immune antiserum for the control. For validation reactions anti-Pc and anti-Pho were used for the ChIP and enrichment was assayed using PCR with specific primers as described previously [55]. The primer sequences are given in Table S4.

### Probe Preparation and Microarray Hybridisation on *Drosophila* Tiled Genomic Microarrays

Three biological replicates were used for each condition and enrichment profiles were generated by comparison of specific and control ChIP DNA samples. In order to identify regions bound by Pho or Pc, 10–20 ng of ChIP and control DNA samples were amplified using a random-primed PCR method according to Affymetrix recommendations (Affymetrix Chromatin Immunoprecipitation Assay Protocol; http://www.affymetrix.com/support/technical/manuals.affx). Purified DNAs were then fragmented, TdT labeled, and hybridized to the Affymetrix *Drosophila* genome Tiling Array 1.0 (reverse part no. 520,054) as described previously [56]. ChIP–array data have been deposited in the GEO database under accession code GSE11006.

### Affymetrix CEL File Analysis

Affymetrix CEL files were converted into chromosomal enrichment profiles using the TiMAT2 package (http://bdtnp.lbl.gov/TiMAT/TiMAT2/). Probe mapping information (“bpmap”) to *D. melanogaster* genome release 4 was obtained from David Nix. Each CEL file was visualised for manual inspection and artefacts were masked using CelMasker. Normalisation was subsequently performed with CelProcessor using default parameters. Enrichment profiles were generated using ScanChip, outputting windowed enrichment signals and Wilcoxon Rank Sum scores. The .sgr files are provided in Datasets S2–5).

### Identification of Bound Regions

We classified enrichment events into positive, intermediate and negative based on visual inspection in the Integrated Genome Browser (http://www.affymetrix.com/support/ developer/tools/download_igb.affx). We found that our manual classification could be automated using basic descriptive statistics. Positive bound regions (“peaks”) were characterised by enrichment values greater than an experiment-specific cutoff as well as a Wilcoxon Rank Sum score greater than 55. Intermediate regions were score-independent but showed an enrichment value greater than 50% of the experiment-specific cutoff. Negative regions accounted for all regions that did not fulfil these criteria. The experiment-specific cutoffs were empirically determined as the signal average plus three standard deviations for Pc (log2 enrichment ratio of 0.39 for the embryo and 0.77 for the T3 disc material) or five standard deviations for Pho (log2 enrichment ratio of 0.62 for the embryo and 0.80 for the T3 disc material). For the S2 Pc data of [10], we followed a similar classification with positive regions having enrichments greater than the signal average plus three standard deviations and negative regions showing ratios of less than 50% of this value.

### Target Gene Assignment

We assigned each binding event to a target gene, based on complete or partial overlap with a gene model. Binding events that did not overlap with a gene model were assigned to the nearest gene. In most cases for Pc, this concerned bound regions that represented extensions of larger domains overlapping with the gene.

### Motif Searches

We selected the 150 strongest Pho peaks that overlapped with Pc binding and generated two different search sets comprising 200 nt or 700 nt of sequence around the centre of the peaks. We used NestedMICA [57] to search for statistically overrepresented sequence motifs in the search set. A first round of searches was performed with NestedMICA v0.72, specifying expected motif length and usage frequency. A second search was performed with NestedMICA v0.8 using default usage frequency and dynamic motif length. Both searches aimed to identify 10–15 overrepresented motifs. Candidate motifs were visually inspected in MotifExplorer and a set of promising candidates resembling Pho-, GAGA- or Zeste-like motifs as well as some with high information content were chosen for further analysis.

Statistical overrepresentation of motifs was determined by comparing the set of all Pho peak sequences to 1,000 sets of random sequences of the same length, representing regions of the *Drosophila* genome that are not bound by Pc. A Z-score was derived, incorporating the number of occurrences in real peaks and the numbers observed for the 1,000 random sets.

All downstream analyses were performed with custom-made Perl scripts. Clustering and visualisation was performed with Genesis v1.6 [58]. Binding sites in the sequence context were visualised in BioSAVE [59].

### Gene Ontology and Tissue Expression Analysis

Enrichment of Gene Ontology terms was determined with the GeneMerge 1.2 software tool, comparing enrichment in specific lists with all *Drosophila* genes. Gene Association files used were March 2007 release of the Gene Ontology. The enrichments quoted in the text are corrected for multiple testing by applying a modified Bonferroni method within the Gene Merge algorithm. Enrichments with e-scores better than 0.05 are called significant. Tissue expression analysis used the data from FlyAtlas [39] with clustering and visualisation using Cluster [60] and Java Treeview [61].

### RT-PCR

OregonR embryos (0–20 hr) were dechorionated with bleach, then divided into aliquots, placed directly in Trizol and stored at –80°C. Homogenisation and RNA extraction were carried out according to the following protocol: http://www.flychip.org.uk/protocols/gene_expression/standard_extraction.php. T3 leg and haltere discs were dissected from wandering 3rd instar OregonR larvae in PBS. Each pair was transferred in a small drop of PBS directly into 100 µl Trizol and frozen immediately. For RNA extraction, 7 disc pairs were randomly pooled for each of 3 samples and RNA extracted as above. RNA samples were treated with RQ1 DNase to remove any genomic DNA. cDNA synthesis was performed by combining 10 µg DNase treated RNA with 500 ng anchored oligo(dT)23 primer (Sigma; Cat. No. 04387), 1 µl of 10 mM dNTPs, DEPC treated H2O to 13 µl. The reaction was heated to 65°C for 5 min then chilled on ice for 1 min. 4 µl of 5x First Strand Buffer (Invitrogen), 1 µl 0.1 M DTT (Invitrogen), 1 µl RNAsin (Promega; Cat. No. 18064-014) and 1 µl Superscript III Reverse Transcriptase (Invitrogen; Cat. No. 18080-044) were added. Reactions were incubated at 50°C for 60 min and inactivated at 70°C for 15 min.

0.5 µl of the resulting cDNA was used in PCR reactions with the following primers: hb-F ggcctcttcgttcacatgg, hb-R agcggcttaattggcttatg, ind-F aacgattatgccgattccag, ind-R gattgaaggtgggactttcg, vnd-F cgacgagatgtcctcgtacc, vnd-R ctcttgtaatcgccggaaag, fd59A-F ttcagtcaccgcacaagaag, fd59A-R gtccagaagttgccctttcc, run-F agtccttcacgctgaccatc, run-R gtagtccgcatagccgtagg, tll-F tacaacagcgtgcgtctttc, tll-R ttgtccaccacacagagtcc, Rp49_F catacaggcccaagatcg, Rp49_R tgggcatcagatactgtcc.

### Accession Numbers

The Flybase (http://flybase.bio.indiana.edu) accession numbers of the genes and gene products discussed in this paper are: *abdominal-A (abd-A),* FBgn0000014*; Abdominal-B (Abd-B),* FBgn0000015*; Antennapedia (Antp),* FBgn0000095*; bagpipe (bap),* FBgn0004862*; biniou (bin),* FBgn0045759*; brahma (brm),* FBgn0000212*; Centrosomal protein 190kD (Cp190),* FBgn0000283*; Cyclin A (CycA),* FBgn0000404*; Deformed (Dfd),* FBgn0000439*; Distal-less (Dll),* FBgn0000157*; dRing (Sce),* FBgn0003330*; GAGA factor (Trl),* FBgn0013263*; GATAe,* FBgn0038391; *gcm2,* FBgn0019809*; glial cells missing (gcm),* FBgn0014179*; grainy head (grh),* FBgn0004586*; hedgehog (hh),* FBgn0004644*; hunchback (hb),* FBgn0001180*; intermediate neuroblasts defective (ind),* FBgn0025776*; labial (lab),* FBgn0002522*; lozenge (lz),* FBgn0002576*; PDGF- and VEGF-receptor related (Pvr),* FBgn0032006*; pleiohomeotic (pho),* FBgn0002521*; pleiohomeotic like (phol),* FBgn0035997*; Polycomb (Pc),* FBgn0003042*; polyhomeotic distal (ph-d),* FBgn0004860*; polyhomeotic proximal (ph-p),* FBgn0004861*; Posterior sex combs (Psc),* FBgn0005624*; proboscipedia (pb),* FBgn0051481*; runt (run),* FBgn0003300*; Scm-related gene containing four mbt domains (Sfmbt),* FBgn0032475*; serpent (srp),* FBgn0003507*; Sex combs reduced (Scr),* FBgn0003339*; suppressor of Hairy wing (su(Hw)),* FBgn0003567*; tailless (tll),* FBgn0003720*; teashirt (tsh),* FBgn0003866*; trithorax (trx),* FBgn0003862*; Ultrabithorax (Ubx),* FBgn0003944*; u-shaped (ush),* FBgn0003963*; ventral nervous system defective (vnd),* FBgn0003986*; vestigial (vg),* FBgn0003975*;* and *zeste (z),* FBgn0004050.

## Supporting Information

Figure S1 Validation PCRs. Fragments across the range of positive, intermediate and negative enrichment on the microarrays were tested for ChIP enrichment by PCR. The positives are generally validated, the intermediates are a mixture with some fragments showing enrichment and the negatives show no enrichment. This validates the chosen thresholds. Also these ChIP assays, based on immunopurification with anti-Pc antiserum, provide validation of the Pc-GFP approach used in the microarray experiments. The primer sequences are given in Table S4.
(3.73 MB EPS)

Figure S2 Examples of motif clusters at Pho binding sites. Representative motif distributions in Pho-bound regions. (1) A peak only comprising the Pho motif. (2) Occurrence of Pho-, Zeste- and GAGA-type motifs in close proximity. (3) TGGCC motifs, and GAGA and Zeste sites. (4) Occurrences of the CGCACT-type motif. (5) A Pho-bound region without canonical Pho motif. Note that the majority of motifs occur around the central region of the peak.
(0.65 MB EPS)

Figure S3 Motif counts at Pho peaks across the BX-C. “Pho peaks” show the peaks above threshold. “Pho motif counts” shows the number of occurrences of the Pho_6 motif (>SOB score -1) in 2 kb windows centred on each peak. “Other motif counts” show the total occurrences of motifs Zeste_12, TGGCC_14, GAGA_14c, CGCACT_7, GAGA_14a, GAGA_6, GTT_10, Zeste_7 (using thresholds given in Table S2) in 2 kb windows centred on each peak. There is considerable variation in motif counts for different peaks and characterised PREs do not stand out as particularly motif-rich.
(0.68 MB EPS)

Figure S4 Comparison with the data of Negre et al. The matrix diagram shows the relationship between Pc targets identified in our analysis and the PRC1 targets identified in the study by Negre et al. [12] in the 3 Mb Adh region. For the Negre et al. data we follow their target criteria: a FDR less than 10% for both Pc and Ph. Targets for the pupal stage are not plotted as, for this stage, only Pc FDR data are available. The first two columns show our Pc targets for the T3 imaginal disc and embryo chromatin, the last three columns show the Negre et al. PRC1 targets for embryo, adult female and adult male chromatin. Our data show very similar profiles of Pc binding between embryo and disc chromatin with only one gene showing differential binding. The Negre et al. embryo data show considerable overlap with our data; 5 out of 7 of our embryo targets are also identified in the Negre et al. data. The adult profiles are more divergent; however we note that two genes, CG4218 and osp, that are only bound in the adult samples in the Negre et al. data are identified as targets in our embryo data, suggesting that they are unlikely to be truly differential targets. As pointed out by Negre et al. the adult male chromatin appears to have a markedly different profile of Pc binding than the other samples.
(0.41 MB EPS)

Figure S5 FlyAtlas cluster diagram.
(1.44 MB EPS)

Table S1 Summary of Pc and Pho binding by gene. Table shows the occupancy calls for genes (using FBgn identifiers and gene symbols) for Pc and Pho in the ChIP data for embryo, T3 disc and S2 cell chromatin. For S2 cells the analysis is based on data from [10]. “Peak_hit” is above positive threshold and within the transcription unit; “peak_near” is above positive threshold and associated with the nearest gene; “grey_hit” is above the intermediate threshold but below the positive threshold and within the transcription unit; “grey_near” is above the intermediate threshold but below the positive threshold and associated with the nearest gene. The last column provides an FBgn or other identifier for genes identified as Pc targets in Kc cells in Tolhuis et al. [11].
(0.14 MB XLS)

Table S2 Motif thresholds and provenance. Sub-optimal bit score thresholds for the 18 motifs and the NestedMICA parameters used for the different motifs.
(0.02 MB XLS)

Table S3 GO analysis for differentially bound Pc targets.
(0.04 MB DOC)

Table S4 Validation Primers.
(0.02 MB XLS)

Dataset S1 Motifs. Motif descriptions are given in .xms format as output by NestedMICA for viewing using MotifExplorer from the NestedMICA package.
(0.06 MB TXT)

Dataset S2 Pc embryo binding profile. Windowed enrichment ratios (log2Ratios) for Pc ChIP on embryo chromatin.
(34.21 MB ZIP)

Dataset S3 Pho embryo binding profile. Windowed enrichment ratios (log2Ratios) for Pho ChIP on embryo chromatin.
(34.23 MB ZIP)

Dataset S4 Pc T3 imaginal disc binding profile. Windowed enrichment ratios (log2Ratios) for Pc ChIP on T3 disc chromatin.
(34.14 MB ZIP)

Dataset S5 Pho T3 imaginal disc binding profile. Windowed enrichment ratios (log2Ratios) for Pho ChIP on T3 disc chromatin.
(34.23 MB ZIP)
