# Supplementary material for: Stability and Dynamics of Polycomb Target Sites in Drosophila Development
Source: PLoS Genet. 2008 Sep 5;4(9):e1000178. doi: 10.1371/journal.pgen.1000178 (PMC2525605; doi:10.1371/journal.pgen.1000178)
Supplement: Table S3 — GO analysis for differentially bound Pc targets. (0.04 MB DOC) [file pgen.1000178.s008.doc]

|  | e-score | Description |
| --- | --- | --- |
| **Pc-bound in embryos but not in T3 discs**  **49 genes** | | |
|  | 3.01E-9 | regulation of transcription from RNA polymerase II promoter |
|  | 1.22E-4 | neuroblast fate determination |
|  | 3.70E-4 | ventral cord development |
|  | 2.55E-03 | regulation of transcription, DNA-dependent |
|  | 9.97E-3 | central nervous system development |
|  | 1.04E-02 | ectoderm development |
|  | 2.92E-02 | regulation of transcription |
|  | 4.66E-02 | pattern specification process |
| **Pc-bound in embryos but not in S2 cells**  **107 genes** | | |
|  | 4.20E-09 | regulation of transcription from RNA polymerase II promoter |
|  | 9.00E-07 | regulation of transcription |
|  | 1.65E-5 | ectoderm development |
|  | 1.66E-5 | sensory organ development |
|  | 2.62E-5 | nervous system development |
|  | 5.11E-5 | cell fate specification |
|  | 3.53E-3 | terminal region determination |
|  | 4.10E-03 | regulation of transcription, DNA-dependent |
|  | 1.01E-2 | Bolwig's organ morphogenesis |
|  | 1.01E-2 | catecholamine metabolism |
|  | 1.18E-2 | synaptic target recognition |
|  | 1.84E-2 | antennal morphogenesis |
|  | 2.53E-2 | torso signalling pathway |
|  | 3.51E-2 | ectoderm-mesoderm interaction |
|  | 3.51E-2 | optic placode development |
|  | 4.37E-2 | Notch signalling pathway |
| **Pc-bound in T3 discs but not in S2 cells**  **119 genes** | | |
|  | 2.70E-7 | Notch signaling pathway |
|  | 4.37E-5 | sensory organ development |
|  | 9.90E-5 | nervous system development |
|  | 3.52E-4 | eye development (sensu Endopterygota) |
|  | 3.57E-3 | ectoderm development |
|  | 8.70E-3 | olfactory learning |
|  | 1.77E-2 | Bolwig’s organ development |
|  | 2.78E-2 | peripheral nervous system development |
|  | 3.20E-2 | antennal morphogenesis |
